# Dietary association of iron deficiency anemia and related pregnancy outcomes

**DOI:** 10.1002/fsn3.2373

**Published:** 2021-06-21

**Authors:** Hina Zulfiqar, Imran Ullah Shah, Muhammad Naveed Sheas, Zahoor Ahmed, Umaira Ejaz, Irfan Ullah, Saad Saleem, Muhammad Imran, Muhammad Hameed, Banaras Akbar

**Affiliations:** ^1^ Department of Diet & Nutritional Sciences University of Lahore Islamabad Campus Pakistan; ^2^ Human Nutrition and dietetics (HND) Iqra University North Campus Plot no. 204 _ 205, Sector 7B/1, North Karachi Karachi Pakistan; ^3^ School of Food Science and Engineering South China University of Technology Guangzhou China; ^4^ Key Laboratory of Resource Biology and Biotechnology in Western China College of Life Sciences North West University Xian Shaanxi China; ^5^ Department of Physical Therapy Iqra University Karachi Pakistan; ^6^ Department of Pharmacy Iqra University Karachi Pakistan; ^7^ Department of Water Management Faculty of Crop Production The University of Agriculture Peshawar Peshawar Pakistan; ^8^ Medical Faculty of Paktia University Paktia Afghanistan

**Keywords:** birthweight, diet, iron deficiency anemia, pregnancy, public health

## Abstract

Iron deficiency anemia (IDA) is a common public health occurrence of pregnancy which is particularly prevalent in developing countries like Pakistan. During this critical period, the deficiency of iron is very common as the iron requirements are greatly enhanced. IDA during pregnancy is associated with intrauterine growth retardation, premature birth, low birthweight, increased labor time, higher risk of infection, elevated maternal and prenatal mortality, muscle dysfunction, and low physical capacity. The present study was aimed to check the prevalence, effect of diet on IDA and its association with the pregnancy outcome, that is, birthweight. Five different public sector hospitals of Rawalpindi and Islamabad were analyzed. A sample size of 500 pregnant females of third trimester was observed which were then followed after delivery from September 2020 to January 2021. A well‐designed questionnaire was developed where different demographic factors, dietary recalls, biomarkers, and other determinants were obtained. The data from the questionnaires were later coded for the purpose of analysis in the statistical package (SPSS) using chi‐square test. Our study indicated that IDA is a moderate public health problem among pregnant women in twin cities and more than half of study subjects have depleted iron stores. Approximately 63% of the subjects were having iron deficiency and 24.8% were facing severe iron deficiency anemia. Only 12% of the subjects were considered as normal. The high prevalence of anemia in our subjects was probably due to low iron intake and poor dietary habits. The results of the study may not be generalizable due to time constraints and other factors. Maternal anemia still remains a major public health concern that requires more attention especially in developing and underdeveloping countries.

## Graphical abstract

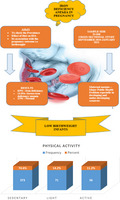

## 1 INTRODUCTION

Iron deficiency anemia is the most commonly known disorder affecting one‐third of the world's population. It has many consequences particularly in the pregnant females indicating that the overall physiologic adaptations are not sufficient to fulfill the nutritional needs (Garzon et al., 2020). World Health Organization (WHO) has estimated over 2 billion of population as being anemic. It is the cause of billions of death each year across the whole globe. It is a major public health issue affecting 41%–50% of the pregnant females in the world. It affects not only developing but also developed countries. But it is mostly prevalent in African countries (Sukrat et al., 2013; Zimmer et al., 2020).

Nearly 18% of the pregnant females living in developed countries and 56% of the women living in developing countries were facing anemia during pregnancy (Sekhavat et al., 2011). More than half of the deaths belonged to only South Asian countries. Of them 80% of the deaths were contributed by India alone (Soundarya & Suganthi, 2017). In Pakistan, the prevalence of anemia among ever‐married women aged 15 to 44 was reported to be 26% in urban areas and 47% in rural areas (Din et al., 2019). The prevalence of anemia among pregnant women living in urban areas was also similar, ranging from 29% to 50% among pregnant women attending antenatal clinics in a large private, tertiary hospital in Karachi (Baig‐Ansari et al., 2008).

Iron deficiency anemia, one of the main types of anemia, is a condition in which the blood of an individual suffering with it lacks a sufficient number of red blood cells. The role of red blood cells is critical in transport of oxygen to the body tissue of an individual for the efficient functioning. Such a condition develops in a patient due to the insufficient amount of iron in the blood.

Iron is a mineral which is essential for the production of red blood cells (RBCs) in the body. Reduced levels of iron lead toward its deficiency and ultimately iron deficiency anemia. If the body has diminished supply of iron, then it means that it also has decreased production of RBCs. The deficiency of iron might be the result from the decreased dietary iron, and there may be problem in the absorption or the iron may be less bio‐available. It may also occur as a result of diminished iron stores in the body, increased loss of iron through bleeding or increased demands for iron, for example, in pregnancy (Short & Domagalski, 2013).

During pregnancy iron deficiency anemia may be diagnosed if the hemoglobin concentrations fall below 10 g/dl and is associated with suboptimal fetal outcome, low birthweight, and preterm delivery along with increased risk of maternal morbidity and mortality (Levy et al., 2005) During 6–12 weeks of gestation, maternal plasma volume increases by 10%–15% and continues to increase until 34 weeks. Nearly 50% of the plasma volume increases in successful pregnancies. 20%–30% of RBCs volume increases till the end of pregnancy if the women are maintaining their iron levels.

The diagnostic tests for identification of iron deficiency anemia include serum ferritin, serum iron concentration, transferrin saturation, total iron binding capacity, and complete blood count. Serum ferritin concentration is the best known test for its diagnosis (Nawsherwan et al., 2020).

The deficiency of iron‐deficient anemia has been recognized mostly due to the nutrition deficit within the individuals. The risk of such deficiency is increased in the early years of childhood as well as during the pregnancy time periods among women. It is due to the fact that in these time periods the growth requires more iron as compared to other time periods.

Keeping in view the importance of the concept, the underlying study attempted to investigate the situation of IDA in the hospitals of the twin cities of Pakistan. It is very important to accelerate the pace of research in this area in order to deal it according to the contextual issues.

Research objectives of the study include:

- To determine the prevalence of iron deficiency anemia.
- To identify the risk factors related to iron deficiency anemia.
- To evaluate the relationship between hemoglobin levels and pregnancy outcome, that is, birthweight.
- To ascertain the effect of diet therapy on management of iron deficiency anemia.

The purpose of this study is also to assess females who are expecting and are currently anemic and understand how their dietary and lifestyle choices lead to their condition and how with proper nutritional interventions it can be managed to reduce the risk of both maternal and infant‐related complications.

### 1.1 Problem identification

The purpose of this study was also to assess the iron deficiency anemia status among the pregnant females in the twin cities of Pakistan, that is, Islamabad and Rawalpindi, as well as to understand how their dietary and lifestyle factors affect their disease condition and how proper nutritional interventions will be helpful in reducing the risk of both maternal and infant complications. Without any doubt, it is considered as one of the major public health problem not only in the twin cities but also throughout the whole globe. As IDA tends to cause unfavorable conditions to female's health with respect to her pregnancy, not only this, it may also lead to the deficiency of other critical micronutrients as well as poor fetal–maternal outcome.

### 1.2 Problem statement

The prevalence of anemia in women during pregnancy, and its prevention and treatment has been one of the core targets of WHO to deal throughout the world. Still this has been an underevaluated problem, not only in the developing but also in the developed countries as well. The developing countries, however, suffer even more adversely due to the severity, duration, and depleted stores. Moreover, it is also critical to treat its symptoms in the early stages as to prevent the individuals to indulge in moderate or severe forms of anemia.

## 2 MATERIALS AND METHODS

### 2.1 Data collection

Data are collected from five different government sector hospitals of Rawalpindi and Islamabad, that is, Pakistan Institute of Medical Sciences (PIMS), Federal Government Polyclinic Islamabad, Holy Family Hospital, Benazir Bhutto Hospital, and District Hospital Rawalpindi.

The data were collected by visiting each patient individually. The predesigned questionnaire was filled with the help of the file and reports of the patient. The dietary history and recalls were asked by them in presence of one of their family members. However, their present condition was also discussed with their respective healthcare provider.

### 2.2 Project design

The data have been collected according to a predesigned questionnaire. It included the demographic, personal, and dietary information; in addition to this, it also contains the details of medical history. Other questions were their medication history with current medications, nutritional supplements, weight, height, eating habits, tea intake, and physical signs and symptoms. The questions involved in the questionnaire were name, age, place of residence, education of the patient, education of their husband, occupation of the patient, occupation of the husband, monthly income, gestational week, parity, gravidity, age of the last born, medical history, current medical status, LFTs, hemoglobin levels, mean corpuscular volume (MCV), hematocrit and mean corpuscular volume hemoglobin. It also included the blood CP report to evaluate the condition of the patient. Other questions also included were their medication history with current medications, nutritional supplements, weight, height, eating habits, tea intake, and physical signs and symptoms. Questions were also asked related to predesigned food frequency questionnaire. The total number of questionnaires used for the purpose of current study was 500. Out of these 500 questionnaires, 100 are obtained from the respondents of PIMS, 100 from District Hospital Rawalpindi, 100 from Holy Family Hospital Rawalpindi, 100 from Polyclinic Hospital Islamabad, and 100 from Benazir Bhutto Hospital Rawalpindi.

#### 2.2.1 Participants

The participants of this study were pregnant women of third trimester. Pregnant females of 1st and 2nd trimesters were excluded. A total number of 500 females who were pregnant at that time were approached for filling the questionnaire in order to get the real‐time information.

### 2.3 Analytical methods

A structured questionnaire was designed for the purpose of collecting data from the participants of the study. For this purpose, pregnant females visiting the general OPD and admitted in Gynecology ward were interviewed and their file was read to obtain the necessary information. The data regarding the personal factor, sociodemographic history, medical and dietary history such as name, age, education status, occupation, household income, gravid, para, medical history, laboratory values, dietary habits, and lifestyle factors were collected.

#### 2.3.1 Data analysis

After the collection of the data, the next step was to analyze the data so that the meaningful insights could be obtained. For this purpose, a statistical software named SPSS was used. In order to analyze the data, it was initially coded according to the requirements of the software to proceed with the analysis part. Chi‐square test was considered as the most appropriate for this study. This test aims to affirm whether there is significant or nonsignificant association between different factors.

## 3 RESULTS AND DISCUSSIONS

All the raw data were first put on the Microsoft Excel 2016. The data were arranged properly on different sheet with codes 0, 1, 2, 3, 4, etc. All the organized data of 500 patients were then transferred to SPSS ver. 23.0. Here, data were also arranged once again properly in order to analyze the results accurately. With the help of SPSS, the final statistical method chi‐square was applied. This helped to check whether the results of different determinants were associated significantly or nonsignificantly with the hemoglobin levels of the patients.

### 3.1 Distribution of monthly income

**FIGURE 1:**
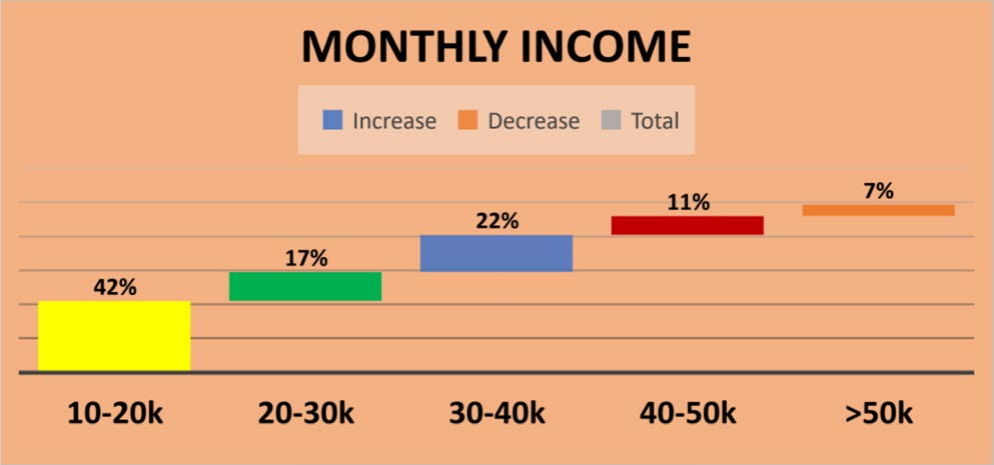
Percentage distribution of monthly income

As most of the females were housewives and their husbands were labors, their monthly income also lies between 10–20 k with 42.2 percentage. Only 6.6% were having their monthly income above 50 k per month as shown in Figure 1. The result of association obtained from chi‐square test shows that there is no association between the economic status of the patients and the prevalence of iron deficiency anemia as the *p*‐value is more than .05, that is, 0.500.

### 3.2 Distribution of type of pregnancy

Table 1 shows that only 5 of the females were having twin pregnancy and all others, that is, 495 were having single pregnancy. From this information, we came to know that majority of females gave birth to only single newborn.

**TABLE 1:** Frequency and percentage distribution of type of pregnancy

Type of pregnancy	Frequency	Percent	Valid percent	Cumulative percent
Single	495	99.0	99.0	99.0
Twin	5	1.0	1.0	100.0
Total	500	100.0	100.0	

### 3.3 Distribution of age of gestation

**FIGURE 2:**
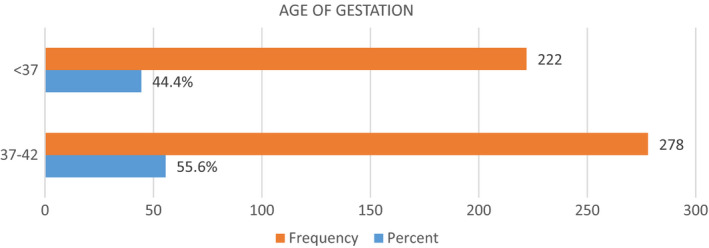
Frequency and percentage distribution of age of gestation

Age of gestation less than 37 weeks was considered as preterm deliveries in the present study. Figure 2 shows that almost 44% of the females gave birth to the infants before 37th week of gestation and 56% gave normal birth to the infants from 37 to 42 weeks. The underlying study results showed that there is statistically no association of gestational age with that of the hemoglobin levels of the pregnant females. As the *p*‐value is >.05, that is, 0.458, this means that our results are nonsignificant and proves no association of gestational week with that of the IDA. However, most of the studies showed a positive correlation preterm deliveries less than 37 weeks with the iron deficiency anemia in pregnancy. The results of the underlying study might vary because of the small sample size or other associated factors.

Srour *et al*. conducted in Palestine with a sample size of 300 pregnant females. The results showed a positive association of maternal anemia condition with the low birthweights with a *p*‐value of .001. The results also go positively in hands with the preterm deliveries having a *p*‐value of .003 (Srour et al., 2018).

### 3.4 Distribution of menstrual bleeding

Menstrual bleeding is also considered as one of the determinants. Most of the pregnant females were having normal bleeding before pregnancy. They were categorized as heavy, normal, light, and very light. Data shown in Table 2 suggest that 74.4% and 13.4% of the females were having light and normal menstrual bleeding before pregnancy, respectively, Only 7.4% and 4.8% were facing very light and heavy menstrual bleeding, respectively, before being expected.

**TABLE 2:** Frequency and percentage distribution of menstrual bleeding

Menstrual bleeding	Frequency	Percent	Valid percent
Heavy	24	4.8	4.8
Normal	67	13.4	13.4
Light	372	74.4	74.4
Very light	37	7.4	7.4
Total	500	100.0	100.0

### 3.5 Distribution physical activity

**FIGURE 3:**
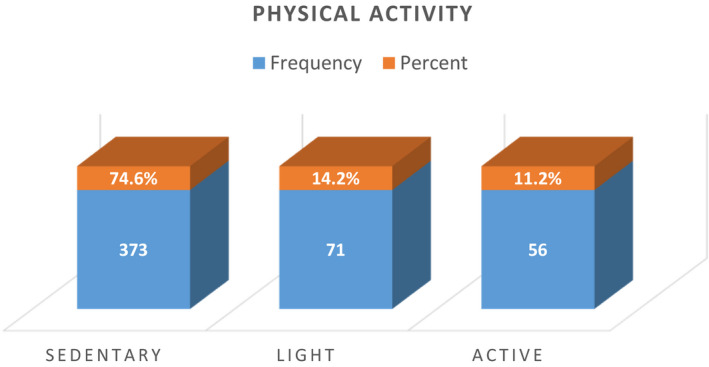
Frequency and percentage distribution of physical activity

Physical activity was one the major determinants. They were categorized as sedentary, light, and active. Figure 3 shows that maximum females belong to the sedentary group, that is, 74.6%. Only 14.2% and 11.2% were doing some kind of activity from light to active, respectively, The *p*‐value is .743 which is >0.05. Hence, this shows that there is statistically no association between physical activity levels with that of the hemoglobin levels. Our results suggest that it does not matter whether you are physically active or not. However, this really depends upon your eating choices as well as physiology of your body.

### 3.6 Distribution of hemoglobin levels

**FIGURE 4:**
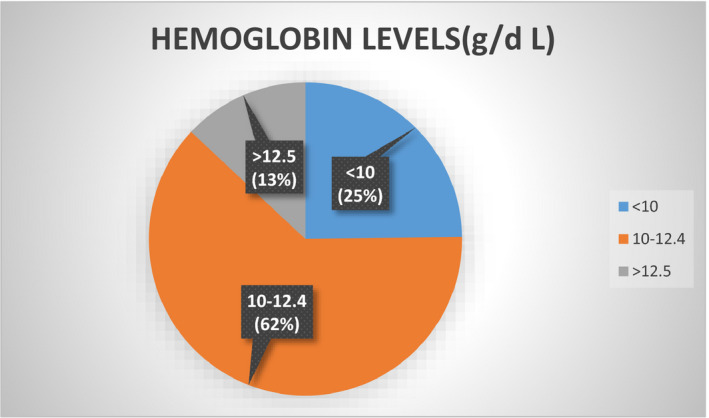
Frequency distribution of hemoglobin levels

24.8% of the pregnant females were having severe anemia with less than 10 g/d L hemoglobin levels. Almost 63% were having iron deficiency and only 12% belonged to the normal hemoglobin levels greater than 12.5 g/d L as shown in Figure 4.

### 3.7 MCV levels versus hemoglobin levels

Mean corpuscular volume of the total blood count is significantly associated with that of the hemoglobin levels, as the p‐value is 0.001. The results indicate that there is a positive association between both these factors, and the results are highly significant as shown in Table 3. Most of the pregnant females having low MCV values are also low in hemoglobin levels. This means that IDA is highly indicated by taking into consideration both the hemoglobin levels and mean corpuscular volume.

**TABLE 3:** Association of MCV Vs hemoglobin levels

MCV Levels	Hemoglobin levels (g/d L)			Total	*p*‐value
	<10	10–12.4	>12.5		
>/84 fl	10	297	71	378	.001
<84 fl	116	0	6	122	
	126	297	77	500	

PJ in 2000 carried out a study that showed the association between mean corpuscular volume and maternal hemoglobin help in indicating iron status levels. Hemoglobin concentration as <95 g/L and MCV as <84 fl represent iron deficiency. The values of hemoglobin between 95 and 105 g/L indicate a very little incidence of neonates of low birthweight and preterm labor (Soundarya & Suganthi, 2017).

### 3.8 Distribution of infants' birthweight

**FIGURE 5:**
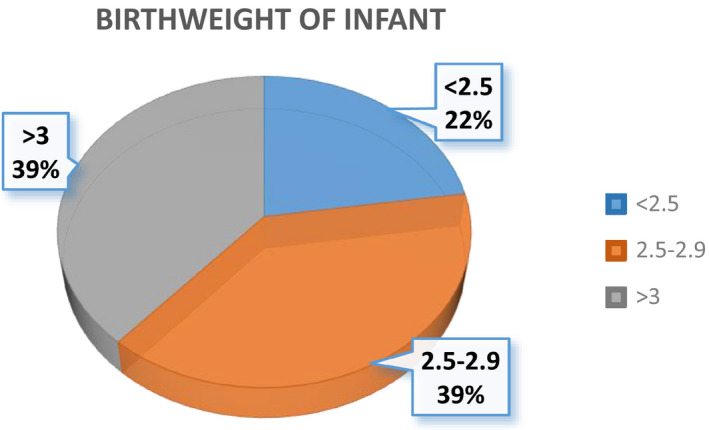
Percentage distribution of infants' birthweight

Figure 5 shows that almost 38.7% of the new born infants belong to the group having moderate birthweights between 2.5–2.9. Similarly, 38.7% are having normal birthweights with 3 or greater. All others were belonged to the group having low birthweights.

The *p*‐value of infants' birthweight found to be 0.001 which is far less than the cutoff points of 0.05. This means that one of the pregnancy outcomes, that is, the birthweight of the infants is significantly associated with the hemoglobin levels of the pregnant females. The women with low hemoglobin levels in pregnancy are expected to deliver infants of low birthweight (Steer, 2000).

A cohort study was carried out at Guangxi during 2014. 500 pregnant females were observed and followed after deliveries. Both the gestational week and birthweights were also observed along with the hemoglobin and ferritin levels. The results show a positive correlation between both mother's anemic status and newly born infant birthweight (Huang et al., 2015).

Hansen *et al*. published a study in 2020 in order to evaluate the prevalence of IDA in different trimesters. It was noted that the prevalence of IDA was higher in the third trimester in comparison with the first and second. This would significantly lead toward the increased status of low birthweight infants. However, it also made the child more susceptible toward anemia in childhood particularly in the first six months of age (Hansen et al., 2020).

### 3.9 Distribution of fruits intake

As the females are pregnant, most of them were taking fruits on daily basis either apple, guava, pomegranate, and other seasonal fruits. According to the study, 63.2% of the females are taking 1–2 servings, 18.8% are having 2–3 servings, 8% are taking 3–4 servings on daily basis, and 10% of them are taking only 1–2 servings of the fruits on weekly basis.

### 3.10 Distribution of vegetables intake

**FIGURE 6:**
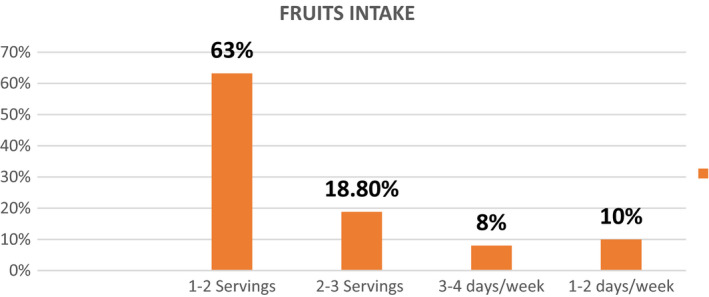
Percentage distribution of fruit intake

**FIGURE 7:**
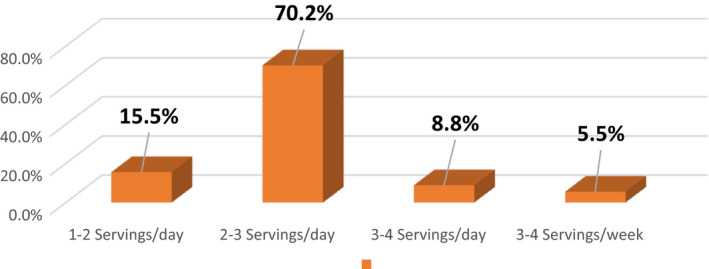
Percentage distribution of vegetable intake

**FIGURE 8:**
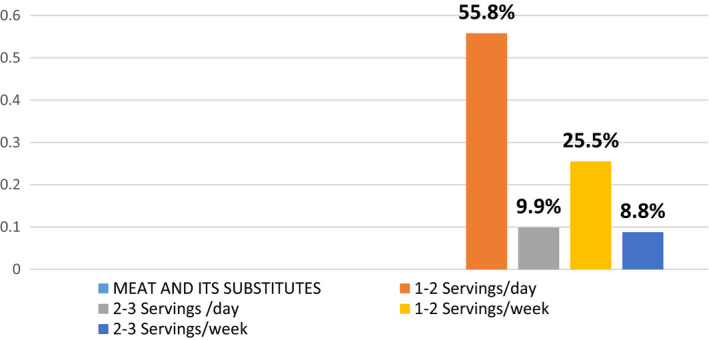
Percentage distribution of intake of meat and its substitutes

Most of the females belong to the poor families, and they mostly consume vegetables on daily basis. Almost 70.2% of the females were taking 2–3 servings of the vegetables per day. 15.5% were taking 1–2 servings of vegetables per day, 8.8% were taking 3–4 servings/day, and 5.5% were having 3–4 servings on weekly basis (Figures 6, 7, 8).

### 3.11 Distribution of meat and its substitutes

The results of our study show that 55.8% of the pregnant females are having 1–2 servings of meat or its substitutes particularly eggs, pulses, legumes, and beans. 25.5% are taking 1–2 servings per week because they were poor or show dislikeness.

## 4 CONCLUSION

Maternal iron deficiency is associated with intrauterine growth retardation, premature birth, low birthweight, increased labor time, higher risk of infection, elevated maternal and prenatal mortality, muscle dysfunction, and low physical capacity. Our study indicated that IDA is a moderate public health problem among pregnant women in twin cities and more than half of study subjects have depleted iron stores. Maternal hemoglobin status was found to affect pregnancy outcome. Newborns born to women with low hemoglobin levels tended to have lower birthweight. Therefore, the etiological factors associated with maternal anemia during pregnancy in Pakistan should deserve more attention.

### 4.1 Limitations of the study

The underlying study was based on the sample from only two cities, Rawalpindi & Islamabad, and focused 5 hospitals only. The results of the study may not be generalizable due to this factor.

### 4.2 Recommendations

In light of the study findings, it is recommended that there is a need to conduct further research on the subject belonging to the developing and underdeveloped nations. So that this major issue will be resolved. This will lead to better policy making and make the right choices. It shall also aid in targeting the grave factor to control this devastating and alarming situation.
